# Inhibitory effect and mechanism of *Lactobacillus crispatus* on cervical precancerous cells Ect1/E6E7 and screening of early warning factors

**DOI:** 10.1186/s13027-023-00483-1

**Published:** 2023-02-01

**Authors:** B. Wan, L. J. Wei, T. M. Tan, L. Qin, H. Wang

**Affiliations:** Gynecologic Tumor Department, Guangxi Medical University Cancer Hospital, 71 Hedi Road, Zhong Shan Street, Nanning, 530021 Guangxi Zhuang Autonomous Region China

**Keywords:** *Lactobacillus crispatus*, Cervical intraepithelial lesions, Co-culture, Differential protein, Early warning factor

## Abstract

**Objective:**

To study the potential mechanism of *Lactobacillus crispatus* inhibiting cervical squamous intraepithelial lesion (SIL) and screen the early warning factors of SIL.

**Methods:**

The effects of *Lactobacillus crispatus* on the proliferation, apoptosis, cross pore migration and invasion and cytokines of cervical precancerous cells Ect1/E6E7 were detected respectively. The effect of *Lactobacillus crispatus* on the expression of differential proteins screened in Ect1/E6E7 cells were detected by Western blot.

**Results:**

*Lactobacillus crispatus* significantly inhibited the proliferation, induced apoptosis and inhibited cell migration of Ect1/E6E7 cells in a time-dependent manner (*P* < 0.05), but had no significant effect on cell invasion. *Lactobacillus crispatus* significantly promoted the secretion of Th1 cytokines and inhibited the secretion of Th2 cytokines by Ect1/E6E7 cells (*P* < 0.05). In addition, compared with SiHa cells in the control group, the expression of differential proteins PCNA, ATM, LIG1 and HMGB1 in Ect1/E6E7cells decreased significantly, while the expression of TDG and OGG1 proteins increased significantly (*P* < 0.05). ABCG2 protein in Ect1/E6E7 cells was slightly higher than that in SiHa cells, but the difference was not statistically significant. What is interesting is that *Lactobacillus crispatus* significantly inhibited the expression of ABCG2, PCNA, ATM, LIG1, OGG1 and HMGB1 proteins in Ect1/E6E7 cells, and promoted the expression of TDG protein.

**Conclusions:**

*Lactobacillus crispatus* may inhibit the function of Ect1/E6E7 cells through multiple pathways and exert the potential to reverse the progression of SIL.

## Introduction

The persistent infection of high-risk human papillomavirus (HR-HPV) is the main pathogenic factor causing cervical squamous intraepithelial lesions (SIL). When the reproductive tract infect HPV, most of it will subside naturally after a period of time, but only a few women have persistent HPV in the vaginal microenvironment and occur SIL after many years. In addition to the factors of HR-HPV persistent infection, the change of microbial community in vaginal microenvironment also plays a very important role in the occurrence of SIL [1]. A variety of microbiome are planted in the vagina, which coordinate and restrict each other to maintain the balance of vaginal microecology. *Lactobacillus* is the most dominant bacterium colonized in the vaginal microecology [1]. It is not only colonized in the vaginal mucosa, but also widely colonized in the oral cavity [2], gastrointestinal tract [3, 4], urinary tract [5, 6], etc. *Lactobacillus* and other microorganisms jointly build mucosal microbial barrier and play an important resistance role in infection, inflammation and tumorigenesis [7].

At present, the mechanism and gene function involved in SIL progression related to vaginal microbial community have not been found. In the early stage [8], our research group used Phylogenetic Investigation of Communities by Reconstruction of Unobserved States (PICRUSt) software to statistically analyze the screened pathways, and the obtained differential pathway information is biologically annotated through Coremine. It is found that there are five differential pathways related to SIL, ABC transporters, Base precision repair, Energy metabolism, Lipid biosynthesis and Protein kinases. The differential genes in five differential pathways were analyzed through bioinformatics, and seven differential genes related to SIL were screened, namely *ABCG2*, *PCNA*, *ATM*, *TDG*, *LIG1*, *OGG1* and *HMGB1* [8].

In this study, *Lactobacillus crispatus*, one of the most common strains of *Lactobacillus* in the vagina of healthy women [9], was selected as the strain used in this study. Ect1/E6E7 cells are human primary cervical epithelial cells transfected with HPV16 E6E7 oncogene into the DNA genome of cervical epithelial cells to achieve cell immortalization [10]. It is considered as a cell model to study cervical precancerous lesions. Therefore, in this study, *Lactobacillus crispatus* and Ect1/E6E7 cells were co-cultured to preliminarily explore the effect and mechanism of *Lactobacillus crispatus* on cervical precancerous cells, so as to provide a new strategy for *Lactobacillus* to prevent and treat SIL.

## Materials and methods

### *Lactobacillus crispatus* and cell line

*Lactobacillus crispatus* (CGMCC: 1.2743) was purchased from China general microbial strain collection center (CGMCC) and anaerobic cultured at 37 °C in MRS medium (Beijing Solarbio Biotech Co., Ltd., Beijing, China). Human cervical precancerous cell line Ect1/E6E7 was purchased from American type culture collection (ATCC; Manassas, VA, USA), and the human cervical cancer cell line SiHa cells were presented by Ma Ruyue who studies in the Ninth People's Hospital Affiliated to Shanghai Jiaotong University. The EV value of the cells presented by Ms. Ma matched with SiHa cells was 1.0 after STR identification. The two cells were cultured in Dulbecco's modified Eagle's medium (DMEM) containing 10% fetal bovine serum (FBS) and 1% penicillin–streptomycin at 37 °C. The reagents used for cell culture were all from Thermo FisherScientific Co., Ltd., Waltham, USA.

### The establishment of co-culture model

Our previous results [11] showed that the effect of *Lactobacillus crispatus* and Ect1/E6E7 cells was the best when the MOI was 200:1. Therefore, to establish the co-culture system model in this study, we chose MOI = 200 as the best multiplicity of infection (MOI) and they were cultured in DMEM without antibiotics but containing 10% FBS.

### CCK-8 assay

Ect1/E6E7 cells (10^4^/100 μl) were seeded in 96-well plates and cultured in DMEM containing 10% FBS, 100U/ml penicillin–streptomycin at 37 °C with 5% CO2. After 24 h of culture, the original culture medium was discarded and gently washed with phosphate buffered solution (PBS) once. The *Lactobacillus crispatus* was resuspended with DMEM. The *Lactobacillus crispatus* with the best MOI was added to each well of the experimental group for 48 h, and divided into groups at an interval of 6 h. Before detection, the original culture medium was discarded and washed with PBS to remove *Lactobacillus crispatus*, and then added 100 μL DMEM and 10 μL CCK-8 reagent (CK04-500 T, Dojindo, Japan) was incubated in 37 °C with 5% CO2 incubator for 2 h, and the optical density of each well was detected at 450 nm.

### Cell apoptosis detection

Ect1/E6E7 cells (10^6^/well) were seeded in 6-well plates and the groups were divided into three groups: control group without any treatment, co-culture experimental group and 30 μg/ml cisplatin (DDP) treated positive control group. The cells of all groups were collected every 6 h. Annexin V and PI were stained at cells of all groups according to the apoptosis kit protocol (556547, BD Pharmingen, USA), and the cell apoptosis was detected by cytoflex flow cytometer (Beckman Coulter, USA).

### Cell migration

Cells were starved 12 h, Ect1/E6E7 cells (2.5 × 10^5^/well) in serum free and antibiotic free DMEM were added to the upper insert of the Transwell chamber (3422, Corning, USA), and *Lactobacillus crispatus* with the MOI = 200 were added to the upper insert. 600 μL DMEM medium containing 20% FBS were added into each hole of the 24 hole plate, and ensure that no bubbles are generated when putting the chamber into a 24 well plate, then put it into the cell incubator for culture. The Transwell chamber at 18 h and 24 h of culture were taken out, then the upper cells were gently wiped with cotton swab, and were washed with PBS twice, and fixed the chamber in a 24 well plate containing 4% paraformaldehyde for 30 min. After taking out the chamber, washed it with PBS for 3 times, dried it naturally, dyed it with crystal violet for 30 min, and washed it with PBS for 3 times. After drying, observation fields were randomly selected under the 100 × microscope for photographing and counting, and three secondary holes were set in each group, the experiment was repeated three times.

### Cell invasion

Cells were starved 12 h, Ect1/E6E7 cells (5 × 10^5^/well) in serum free and antibiotic free DMEM were added to the upper insert of the Transwell chamber (3422, Corning, USA), and *Lactobacillus crispatus* with the MOI = 200 were added to the upper insert. The insert membrane was pre-coated with or without 80 μL Matrigel Matrix (1 mg/ml; Corning, USA). 600 μL DMEM medium containing 20% FBS were added into each hole of the 24 hole plate, and ensure that no bubbles are generated when putting the chamber into a 24 well plate, then put it into the cell incubator for culture. The Transwell chamber at 18 h and 24 h of culture were taken out, then the upper cells were gently wiped with cotton swab, and were washed with PBS twice, and fixed the chamber in a 24 well plate containing 4% paraformaldehyde for 30 min. After taking out the chamber, washed it with PBS for 3 times, dried it naturally, dyed it with crystal violet for 30 min, and washed it with PBS for 3 times. After drying, observation fields were randomly selected under the 100 × microscope for photographing and counting, and three secondary holes were set in each group, the experiment was repeated three times.

### Cytokine detection

Ect1/E6E7 cells (10^6^/well) were seeded in 6-well plates (Corning, USA) and co-cultured with *Lactobacillus crispatus* of MOI = 200. The cell supernatants of the control group and co-cultured group for 6 h, 12 h, 18 h and 24 h were collected, 1000 × *g* centrifuge for 10 min, then the supernatant was collected and frozen in the refrigerator at − 20 °C. Follow the instructions of cytokine detection kit (Wuhan Elabscience, China), the cell supernatants were detected the OD value of each hole at 450 nm, and the average OD value of each concentration gradient of standard hole was calculated, then the calibration value was OD of standard hole subtracted the blank hole, the standard curve with the standard concentration as the abscissa and the calibrated OD value as the ordinate was drawn, and R^2^ of the linear equation should be > 0.99. The sample OD value minus the blank hole is the actual OD value of each sample, which is substituted into the linear equation to calculate the concentration of IL-2, IL-4, IL-10, IL-12 and IFN-γ in each sample.

### Western-blot

Protein expression of seven differential genes of *ABCG2*, *PCNA*, *ATM*, *TDG*, *LIG1*, *OGG1* and *HMGB1* were detected. Cells in the control group and co-cultured group for 6 h, 12 h, 18 h and 24 h were collected, Total cellular protein was extracted using a protein extraction kit (Beijing Solarbio Biotech Co., Ltd., Beijing, China), and protein concentration was measured using the BCA protein kit (Shanghai Beyotime Biotech Co., Ltd., Shanghai, China). The denatured protein samples of 30 μg each were separated on 6% or 8% or 10% or 12% sodium dodecyl sulfate–polyacrylamide gel electrophoresis gels and electrophoretically transferred onto polyvinylidene fluoride membranes (Millipore, USA). For western blot analysis, the membranes were blocked in 5% skimmed milk solution for 2 h and then incubated with a specific primary antibody at 4 °C overnight. The following day, the membranes were washed with 1 × TBST three times and then incubated with HRP binding goat anti-rabbit IgG(H + L) antibody at the room temperature for 2 h. The primary antibodies were rabbit monoclonal antibodies against ABCG2 (catalog no. A1370; ABclonal Technology Co., Ltd., Wuhan, China), PCNA (catalog no. K101310P; Solarbio Biotech Co., Ltd., Beijing, China), ATM (catalog no. K004295P; Solarbio Biotech Co., Ltd., Beijing, China), TDG (catalog no. A5756; ABclonal Technology Co., Ltd., Wuhan, China), LIG1 (catalog no. A1858; ABclonal Technology Co., Ltd., Wuhan, China), OGG1 (catalog no. A2268; ABclonal Technology Co., Ltd., Wuhan, China), HMGB1 (catalog no. H07173023; Wanleibio, Shenyang, China), GAPDH (catalog no. 2118; Cell Signaling Technology, USA) and used at a dilution of 1:1000, while the secondary antibody was an anti-rabbit IgG (catalog no. 7074; Cell Signaling Technology, USA) and used at a dilution of 1:1000. The protein bands were subsequently visualized using chemi-luminescence (Bio-Rad Laboratories, Inc., Hercules, CA, USA) and X‐ray films.

### Statistical analysis

The experimental data are expressed by means ± standard deviation, the data are imported into GraphPad Prism 8 for drawing and statistically analyzed by IBM SPSS statistics 25.0 statistical software. The comparison between the two groups of data adopts independent sample *t* test, and the *t*’-test is selected when the variance is uneven. The data of random block design selects random block design variance analysis, if there are differences, *t* test is selected to further analyze the differences in the group, *P* < 0.05 is regarded as the difference has statistical difference.

## Results

### *Lactobacillus crispatus* inhibits cell growth

**Fig. 1:**
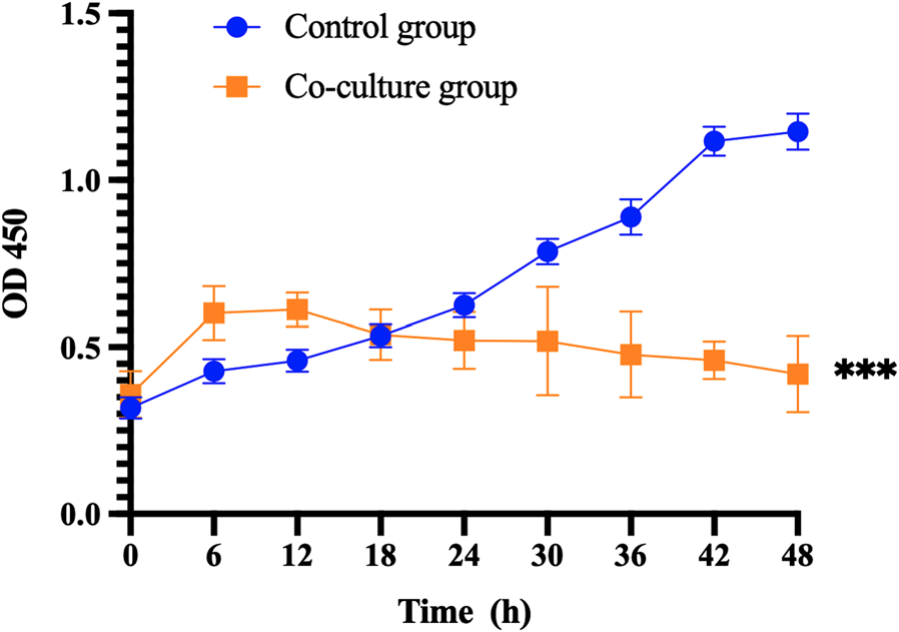
The effect of *Lactobacillus crispatus* on proliferation of Ect1/E6E7 cells. *P < 0.05; **P < 0.01; ***P < 0.001, versus control group

In order to evaluate the anticancer activity of *Lactobacillus crispatus*, the cell proliferation was measured. The results showed that *Lactobacillus crispatus* inhibited the proliferation of cervical precancerous cell line Ect1/E6E7 in a time-dependent manner (Fig. 1).

### Cells apoptosis induced by *Lactobacillus crispatus*

**Fig. 2:**
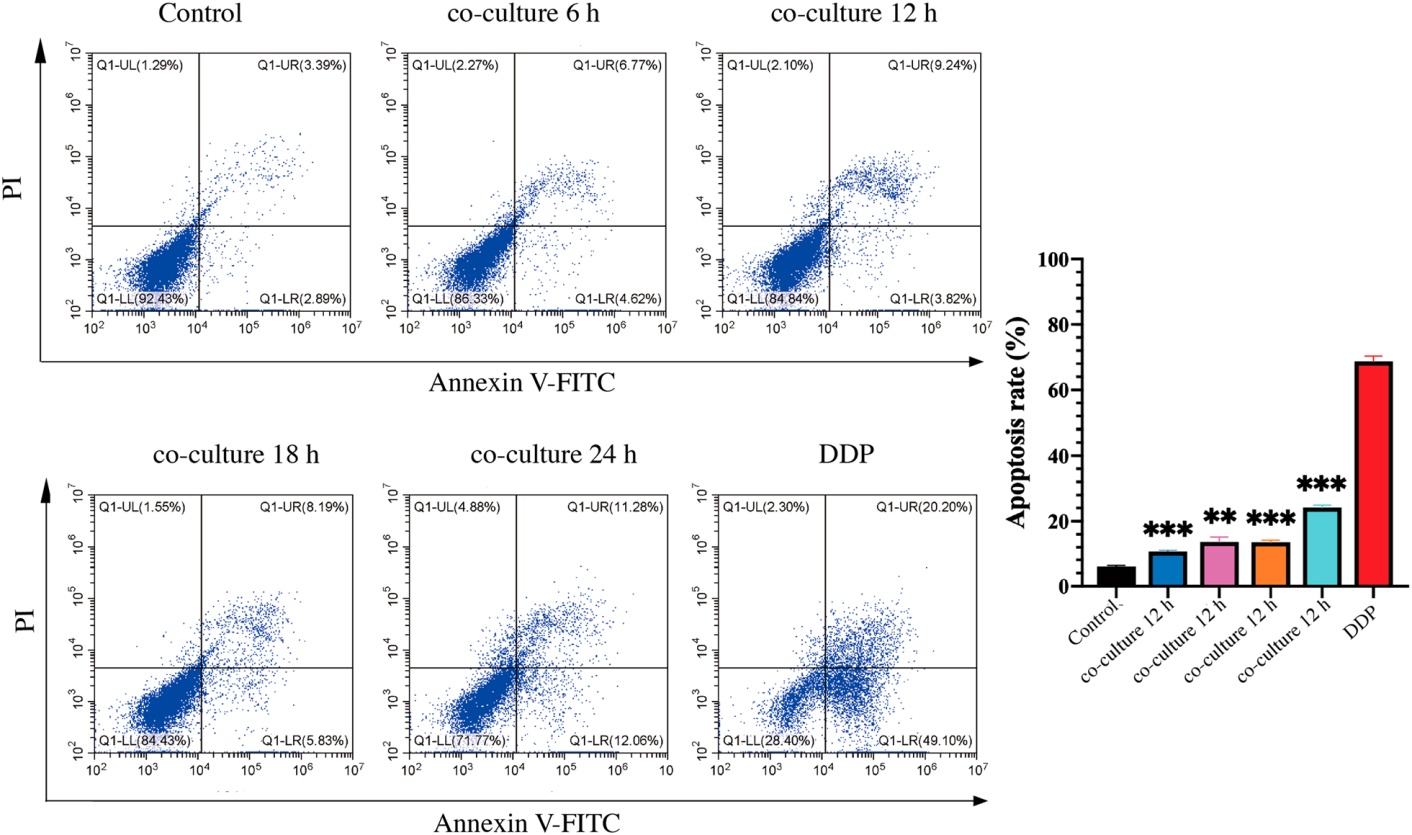
The effect of *Lactobacillus crispatus* on Ect1/E6E7 cells apoptosis. *P < 0.05; **P < 0.01; ***P < 0.001, versus control group

The present study assayed cell apoptosis to evaluate whether the reduction in cell viability was due to the induction of apoptosis. *Lactobacillus crispatus* treatment significantly increased the apoptotic rate of the Ect1/E6E7 cells, compared with the control group (Fig. 2). *Lactobacillus crispatus* mainly induced late apoptosis of Ect1/E6E7.

### *Lactobacillus crispatus* inhibits cell migration and invasion

**Fig. 3:**
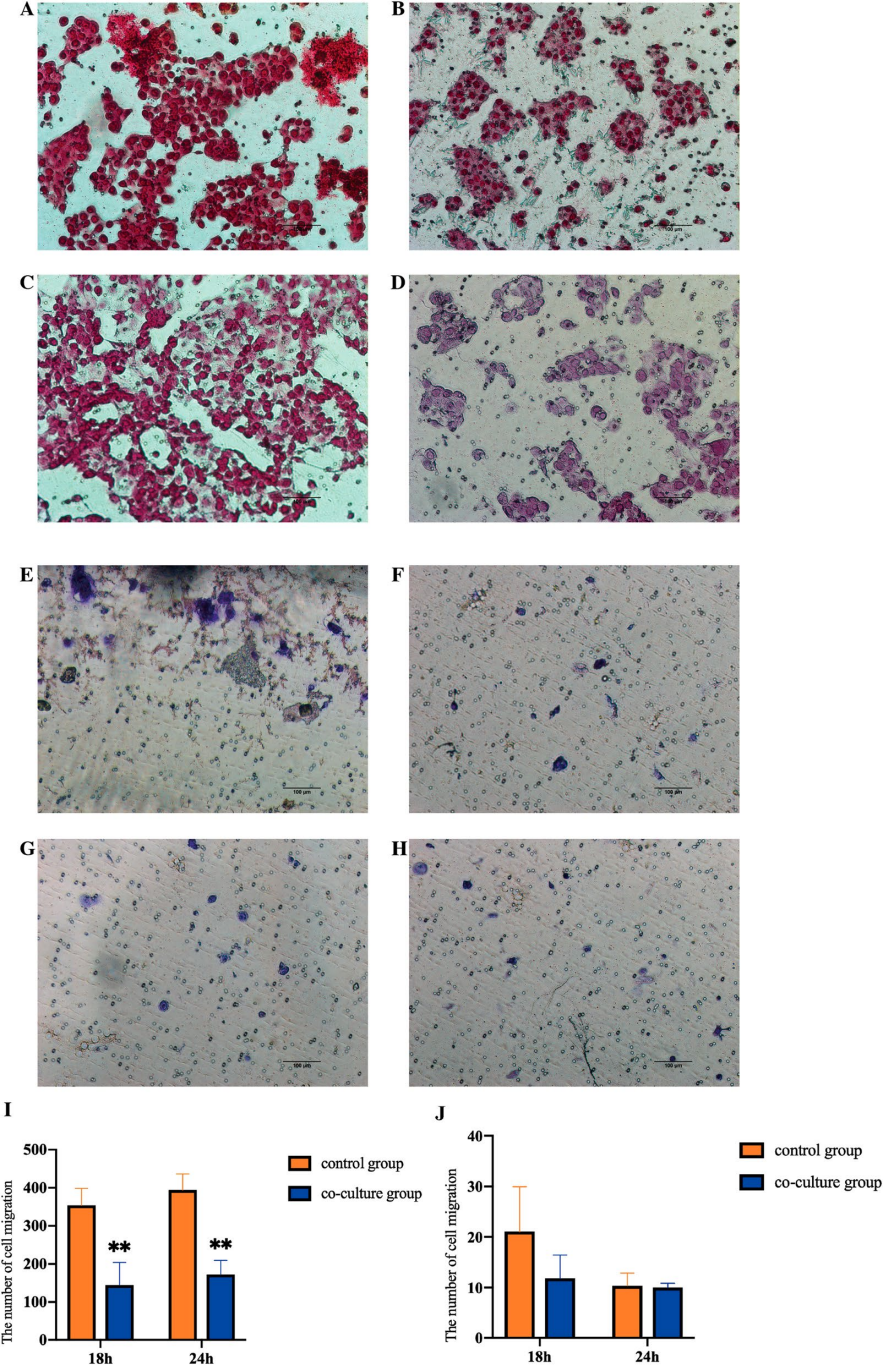
The effect of *Lactobacillus crispatus* on Ect1/E6E7 cell migration and cell invasion. **A** The number of cell migration of Ect1/E6E7 cells at 18 h was measured. **B** The number of cell migration of Ect1/E6E7 cells treated with *Lactobacillus crispatus* for 18 h was observed. **C** The number of cell migration in 24 h of Ect1/E6E7 cells. **D** The number of cell migration of Ect1/E6E7 cells treated with *Lactobacillus crispatus* for 24 h was higher than that of control group. **E** The number of cell migration of Ect1/E6E7 cells at 18 h was measured. **F** The number of cell migration of Ect1/E6E7 cells treated with *Lactobacillus crispatus* for 18 h. **G** The number of cell migration in 24 h of Ect1/E6E7 cells. **H** The number of cell migration of Ect1/E6E7 cells treated with *Lactobacillus crispatus* for 24 h. *P < 0.05; **P < 0.01; ***P < 0.001, versus control group

The results of Transwell chamber test show that *Lactobacillus crispatus* can significantly inhibit the migration ability of Ect1/E6E7 cells (Fig. 3A–D, I), but has no significant inhibitory effect on the invasion of Ect1/E6E7 cells (Fig. 3E–H, J).

### Effect of *Lactobacillus crispatus* on cytokines

**Fig. 4:**
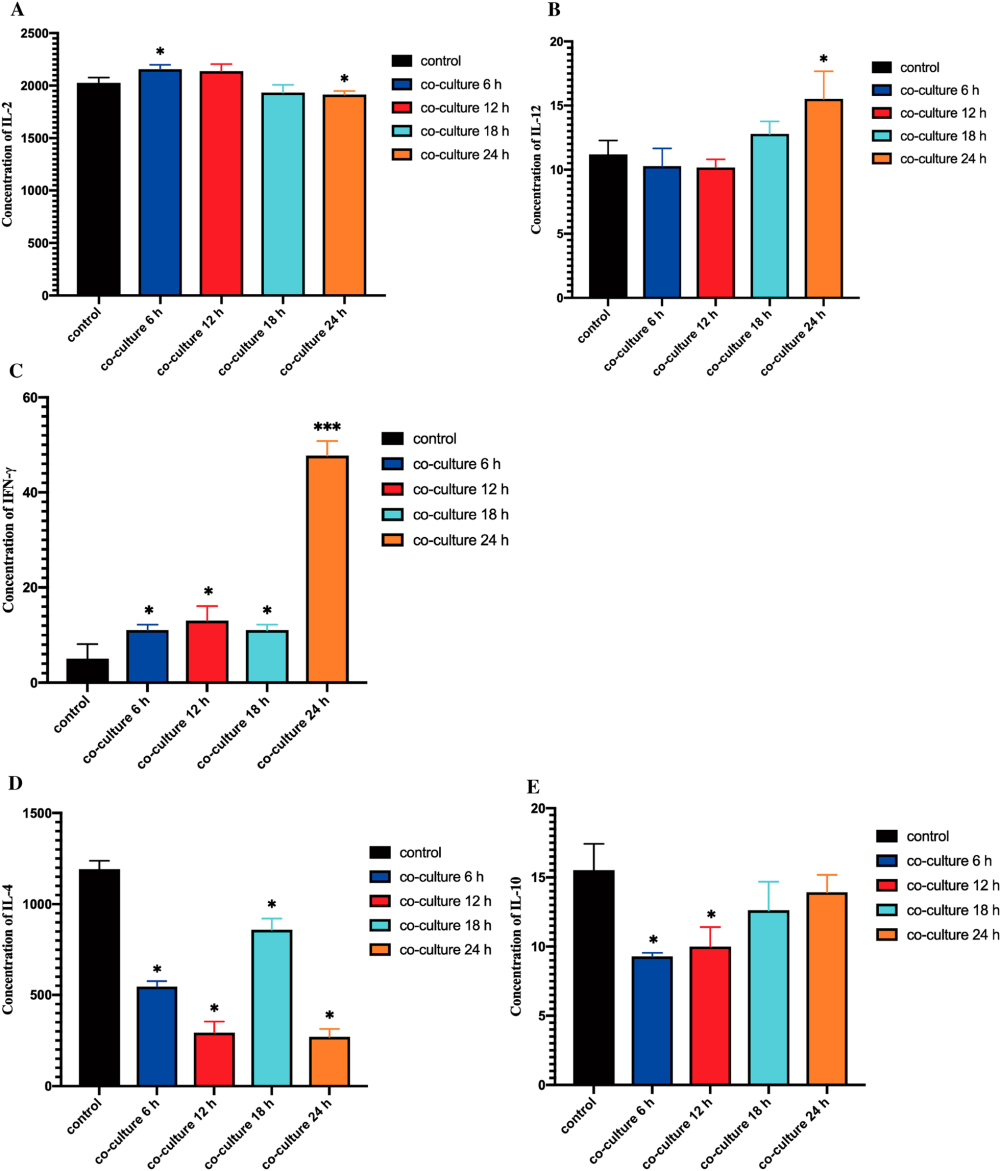
The expression of cytokines in the supernatant of Ect1/E6E7 cells treated with *Lactobacillus crispatus* was detected by ELISA. **A** The expression of IL-2 in the supernatant of Siha cells and Ect1/E6E7 cells treated with *Lactobacillus crispatus* was detected by ELISA. **B** The expression of IL-12 in the supernatant of Siha cells and Ect1/E6E7 cells treated with *Lactobacillus crispatus* was detected by ELISA. **C** The expression of IFN-γ in the supernatant of Siha cells and Ect1/E6E7 cells treated with *Lactobacillus crispatus* was detected by ELISA. **D** The expression of IL-4 in the supernatant of Siha cells and Ect1/E6E7 cells treated with *Lactobacillus crispatus* was detected by ELISA. **E** The expression of IL-10 in the supernatant of Siha cells and Ect1/E6E7 cells treated with *Lactobacillus crispatus* was detected by ELISA. *P < 0.05; **P < 0.01; ***P < 0.001, versus control group

Th1 cytokines are mainly IFN-γ, IL-2, TNF-β, IL-12 and other inflammatory factors and mediate the immune response related to cytotoxicity and local inflammation. Th2 cytokines are mainly anti-inflammatory factors such as IL-4, IL-5, IL-6, IL-10 and IL-13. They can inhibit the production of Th1 cytokines and cytotoxic T lymphocytes (CTL), inhibit antigen presentation, so the body immunity is inhibited. However, there is a shift from Th1 to Th2 in tumor patients, which is more significant with the severity of the disease. In order to further study the mechanism of *Lactobacillus crispatus* inhibiting cell function and inducing apoptosis, the expression of Th1 and Th2 cytokines of Ect1/E6E7 cells treated with *Lactobacillus crispatus* were detected. The results showed that *Lactobacillus crispatus* could significantly promote Ect1/E6E7 cells secreted Th1 cytokines (IL-2, IL-12, IFN-γ), however, it significantly inhibited the secretion of Th2 cytokines (IL-4, IL-10) (Fig. 4).

### Expression changes of differential proteins of Ect1/E6E7 cells treated with *Lactobacillus crispatus*

**Fig. 5:**
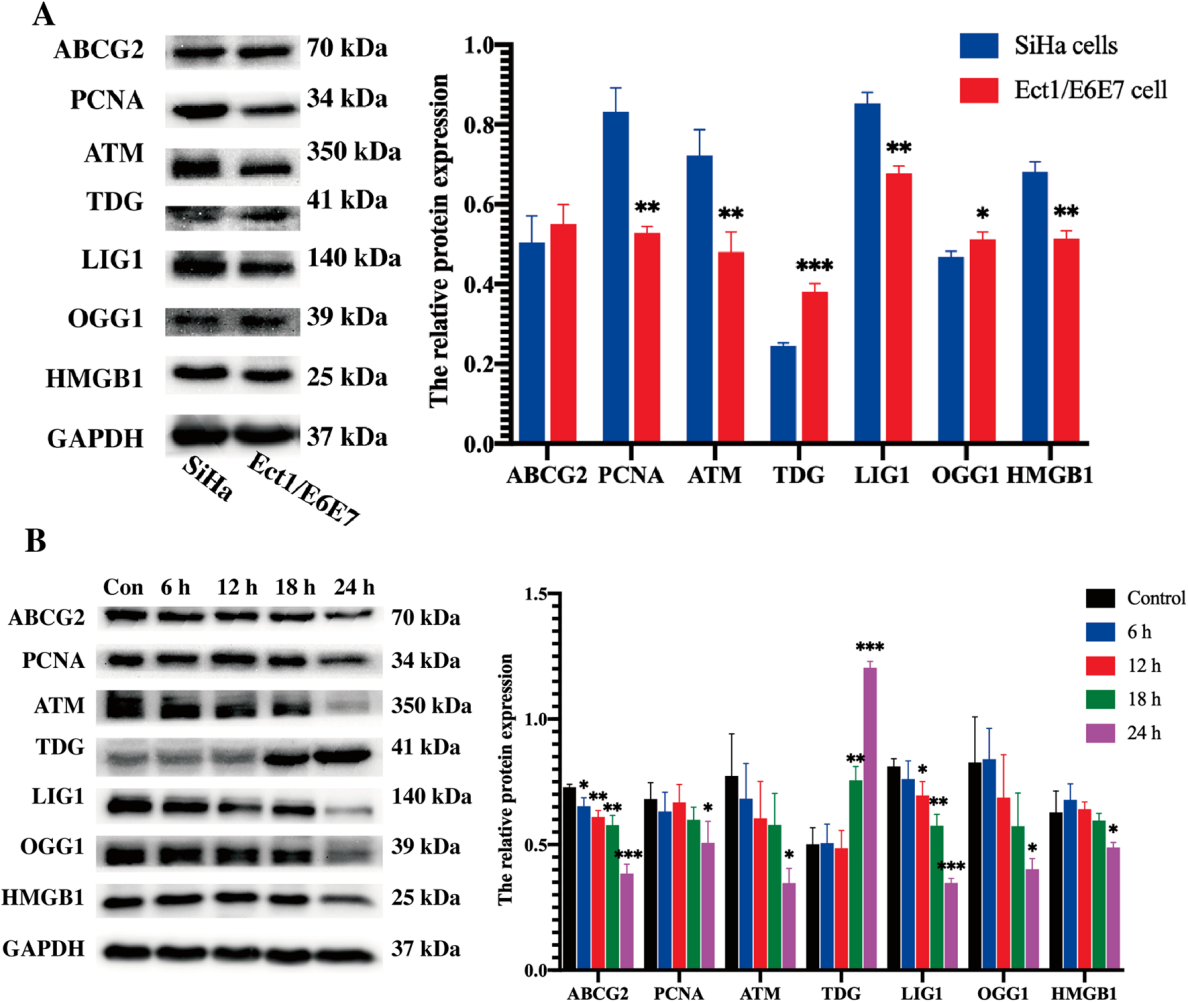
The expression of differentially proteins in Ect1/E6E7 cells and in Ect1/E6E7 cells treated with *Lactobacillus crispatus*. **A** The expression of protein of differentially expressed genes in SiHa cells and Ect1/E6E7 cells. *P < 0.05; **P < 0.01; ***P < 0.001, versus SiHa cells. **B** The changes of protein expression of different genes in Ect1/E6E7 cells treated with *Lactobacillus crispatus*. *P < 0.05; **P < 0.01; ***P < 0.001, versus control group

Western blot was used to detect the protein expression of differential proteins ABCG2, PCNA, ATM, TDG, LIG1, OGG1 and HMGB1 in Ect1/E6E7 cells, and the expression changes of differential proteins of Ect1/E6E7 cells treated with *Lactobacillus crispatus*. The results showed that compared with the control group of cervical squamous cell carcinoma SiHa cells, the expression of differential proteins PCNA, ATM, LIG1 and HMGB1 in Ect1/E6E7 cells decreased significantly, while the expression of TDG and OGG1 proteins increased significantly. ABCG2 protein in Ect1/E6E7 cells was slightly higher than that in SiHa cells, but the difference was not statistically significant (Fig. 5A). After *Lactobacillus crispatus* co-cultured with Ect1/E6E7 cells (Fig. 5B), ABCG2 protein expression decreased significantly at the beginning of 6 h of co-culture, LIG1 protein expression decreased significantly at 12 h of co-culture, PCNA, ATM, OGG1 and HMGB1 protein expression decreased significantly at 24 h of co-culture, and TDG protein increased significantly at 18 h of co-culture. The results of co-culture suggest that *Lactobacillus crispatus* can significantly inhibit the expression of ABCG2, PCNA, ATM, LIG1, OGG1 and HMGB1 proteins but promote the expression of TDG protein in Ect1/E6E7 cells, which may reverse the progress of SIL.

## Discussion

In recent years, studies have found that the change of vaginal microecology is also an important risk factor for SIL and cervical cancer [12, 13]. The imbalance of vaginal microecology can cause genital tract inflammation, vulvar squamous intraepithelial lesion and SIL, long-term persistent HPV infection and cervical cancer [14–18]. Mitra et al. [19] found that the decrease of *Lactobacillus* in vaginal microbiome was closely related to HPV persistent infection and the occurrence of SIL. Other studies found that the detection rate of HPV decreased significantly when the *Lactobacillus* was present [20]. In this study, Ect1/E6E7 cells were used as the cell model of cervical precancerous lesions, which were co-cultured with *Lactobacillus crispatus*. Through a series of experiments, the potential mechanism of *Lactobacillus crispatus* inhibiting SIL and screening related early-warning factors were studied.

### Role and mechanism of *Lactobacillus crispatus* in stabilizing vaginal microecology

*Lactobacillus*, as a dominant bacterium colonized in vaginal mucosa microbiome [9], its cell wall is rich in peptidoglycan, lipoteichoic acid and polysaccharides, in which lipoteichoic acid can make bacteria have good adhesion [21]. There are many studies have found that *Lactobacillus* can adhere to cervical cancer cells and play an adhesion inhibitory effect [22, 23]. Rajoka et al. [24] used the cell-free culture supernatant (CFCs) secreted by *Lactobacillus* isolated from breast milk to act on HeLa cells, and found that CFCs played an anti-cancer role by up regulating the expression of apoptosis genes *BAX*, *BAD*, *Caspase3*, *Caspase8* and *Caspase9* and down regulating the expression of *BCl-2* gene. Wang et al. [25] used *Lactobacillus supernatants* (LS) from *Lactobacillus curlicus*, *Lactobacillus gargari* and *Lactobacillus Jennings* to treat cervical cancer CaSki cells, and found that LS could inhibit the proliferation of CaSki cells, induce cell arrest in S phase, down regulate the expression of HPV *E6* and *E7* oncogenes, *CDK2* and *Cyclina*, and up regulate the expression of *P21*, so as to play an anti-tumor role. The results of this study showed that *Lactobacillus crispatus* can significantly inhibit the proliferation of Ect1/E6E7 cells after 24 h of co-culture (*P* < 0.05). At the same time, *Lactobacillus crispatus* can increase significantly the apoptosis rate of Ect1/E6E7 cells treated with *Lactobacillus crispatus* after 6 h of co-culture (*P* ≤ 0.001). These results suggest that *Lactobacillus crispatus* can inhibit the proliferation of Ect1/E6E7 cells and induce cells apoptosis. Nouri et al. [26] used *Lactobacillus rhamnosus* supernatant (LRs) and *Lactobacillus fragilis* supernatant (LCS) to treat HeLa cells and HT-29 cells and found that both LRS and LCS could reduce the expression of MMMP2 and MMMP9 proteins in HeLa cells and then play a role in inhibiting tumor cell metastasis, while only LCS was effective for HT-29 cells. The results of this study suggest that *Lactobacillus crispatus* can inhibit the migration of Ect1/E6E7 cells. What deserves to be mentioned is only a small number of Ect1/E6E7 cells penetrate the matrix glue and basement membrane, indicating that the matrix metalloprotein degrading matrix glue secreted by Ect1/E6E7 cell is very low, while the effect of *Lactobacillus crispatus* on the invasion of Ect1/E6E7 cells is not significant. It may be that Ect1/E6E7 cells are cervical precancerous cells, which is formed by adding E6E7 oncogene to normal cervical epithelial cells, and its matrix metalloprotein secretion is not affected by *Lactobacillus crispatus*.

### Effect of *Lactobacillus crispatus* on vaginal cytokines

Th1 cytokines are mainly IFN-γ, IL-2, TNF-β, IL-12 and other inflammatory factors. Th2 cytokines are mainly anti-inflammatory factors such as IL-4, IL-5, IL-6, IL-10 and IL-13. In recent years, many studies have found that the subgroup of Th cells in tumor patients are in an unbalanced condition, Th1 drifts to Th2, and it becomes more significant with the severity of the disease, which is manifested in the gradual decrease of Th1 cytokines and the continuous increase of Th2 cytokines [27–29]. Tumor cells can also secrete a variety of cytokines and express a variety of cytokine receptors, which play a very important role in the process of the occurrence and development of tumors. Cervical cancer and other tumors also secrete cytokine IL-2, which plays an immune escape role in the tumor microenvironment and is an essential factor for maintaining the growth of tumor cells. IL-4 and IL-10 are expressed in SIL and cervical cancer, but IL-4 is not expressed in normal cervical tissues. With the progress of cervical lesions, the expression of IL-4 gradually increases and the expression of IL-12 gradually decreases [30–32]. Gruijl et al. [33] found that the expressions of IFN-γ, IL-10 and IL-12 in invasive cervical cancer were significantly lower than those in cervical precancerous lesions, IFN-γ and IL-10 mRNA were associated with the presence of IL-12 p35 and p40 transcripts.

In this study, it was found that Ect1/E6E7 cells in the control group could secrete IL-2, IL-12, IFN-γ, IL-4 and IL-10, and the secretion of IL-2 and IL-4 is large, while IL-12 and IFN-γ and IL-10 were secreted in a small amount. *Lactobacillus crispatus* significantly promoted Ect1/E6E7 cells to secrete Th1 cytokines and inhibited the secretion of Th2 cytokines. The secretion of IL-2 increased significantly at 6 h, and the cell proliferation was promoted at this time, which may be the Ect1/E6E7 cells are precancerous cells, the inhibitory effect of *Lactobacillus crispatus* was not significant and the cells were mainly proliferated at this time. However, IL-2 decreased significantly after 24 h of co-culture, which may be related to a large number of cell death or apoptosis. The secretion of IL-12 increased significantly at 24 h of co-culture, and the cell proliferation began to be significantly inhibited. The secretion of IFN-γ increased significantly at the beginning of 6 h of co-culture. The secretion of IL-4 decreased significantly at the beginning of 6 h. It may be that *Lactobacillus crispatus* caused the continuous decrease of IL-4 secretion by inhibiting the secretion of Th2 cytokines by Ect1/E6E7 cells and promoting the increase of apoptosis. IL-10 secretion decreased significantly at 6 h and 12 h.

### Effect of *Lactobacillus crispatus* on differential genes in cervical microbial community

In the early stage, the differential genes *ABCG2*, *PCNA*, *ATM*, *TDG*, *LIG1*, *OGG1* and *HMGB1* from the vaginal microbiota of SIL patients were screened by our research group. Firstly, the expression of differential gene proteins in the SiHa cells and Ect1/E6E7 cells was found that compared with cervical squamous cell carcinoma SiHa cells, the expression of differential proteins PCNA, ATM, LIG1 and HMGB1 in Ect1/E6E7 cells decreased significantly, while the expression of TDG and OGG1 proteins increased significantly, indicating that *PCNA*, *ATM*, *LIG1* and *HMGB1* may be oncogenes or play a role in promoting cancer, while *TDG* and *OGG1* may be tumor suppressor genes or play a role in inhibiting cancer. ABCG2 protein is also known as breast cancer resistance protein. This study found that the expression of ABCG2 protein in Ect1/E6E7 cells was slightly higher than that in SiHa cells, while other studies reported that the expression of ABCG2 protein in cervical cancer was significantly higher than that in cervical intraepithelial neoplasia (CIN) tissue [34, 35]. PCNA protein is recognized as a marker protein of cell proliferation. The results of this study confirmed that the expression of PCNA protein in Ect1/E6E7 cells was significantly lower than that in SiHa cells, which was consistent with the results of relevant reports [36–38]. ATM protein is a kind of DNA double strand break repair protein. It participates in the precise repair of cells and has a resistance to the destruction of DNA double strand by radiation, so it can be used as a radiosensitivity index of tumor cells. The results of this study confirmed that the expression of ATM protein in Ect1/E6E7 cells was significantly lower than that in SiHa cells, suggesting that SiHa cells have stronger radiation resistance than Ect1/E6E7 cells, while were inconsistent with others reported that there was no significant difference in the expression of ATM protein between cervical cancer and CIN [39]. LIG1 protein is a DNA ligase involved in DNA replication and repair. At present, there is no report of LIG1 protein in cervical lesions. The results of this study confirmed that the expression of LIG1 protein in Ect1/E6E7cells was significantly lower than that in SiHa cells, which was consistent with the results of relevant reports [40]. TDG protein is a key enzyme in the active demethylation of DNA and may be a potential tumor suppressor protein, while there is no report of TDG protein in cervical lesions. The results of this study confirmed that the expression of TDG protein in Ect1/E6E7 cells was significantly higher than that in SiHa cells, suggesting that TDG protein may be a potential tumor suppressor protein or have tumor suppressive effect. OGG1 protein may also be a potential tumor suppressor protein, participating in the base excision repair pathway to repair DNA bases damaged by oxidants or alkylating agents. It may be a potential tumor suppressor protein. The results of this study confirmed that the expression of OGG1 protein in Ect1/E6E7 cells was significantly higher than that in SiHa cells, suggesting that OGG1 protein may also be a potential tumor suppressor protein or have tumor suppressive effect. HMGB1 is a member of the high mobility box superfamily. It participates in DNA replication and repair, regulates transcription, and plays an important role in the process of inflammation, cell differentiation and tumor metastasis. The results of this study confirmed that the expression of HMGB1 protein in Ect1/E6E7 cells was significantly lower than that in SiHa cells, which was consistent with the results reported in relevant [41, 42].

In this study, it was found that the expression of differential proteins ABCG2, PCNA, ATM, LIG1, OGG1 and HMGB1 decreased significantly, while the expression of differential protein TDG increased significantly. It was worth noting that the expression of PCNA, ATM, OGG1 and HMGB1 proteins decreased significantly after co-culture for 24 h. This study suggests that *Lactobacillus crispatus* can significantly inhibit the expression of potential oncogenes *ABCG2*, *LIG1*, *PCNA*, *ATM*, *OGG1* and *HMGB1* in Ect1/E6E7 cells, but up regulate the expression of tumor suppressor gene *TDG*, which may reverse the progress of SIL.

## Conclusion

This study suggests that *Lactobacillus crispatus* can significantly inhibit the proliferation, induce apoptosis and inhibit cell migration of Ect1/E6E7 cells in a time-dependent manner, significantly promote the secretion of Th1 cytokines and inhibit the secretion of Th2 cytokines by Ect1/E6E7 cells, and significantly inhibit the potential cancer promoting genes ABCG2, LIG1, PCNA, ATM, OGG1 and HMGB1 protein expression, but promote the tumor suppressor gene TDG protein expression. These results suggest that *Lactobacillus crispatus* may inhibit the function of Ect1/E6E7 cells through multiple ways and play the potential to reverse the progression of SIL. The early warning factors of SIL include Th1 cytokine, Th2 cytokine and differential protein ABCG2, LIG1, PCNA, ATM, TDG, OGG1 and HMGB1 in this study.

## Data Availability

The datasets used and/or analyzed during the current study are available from the corresponding author on reasonable request.
